# Bilateral adrenal hemorrhage: learning notes from clinical practice and literature review

**DOI:** 10.3389/fendo.2023.1233710

**Published:** 2023-11-03

**Authors:** Maria Elena Aloini, Sara Manella, Irene Biondo, Roberta Maggio, Guido Roberto, Francesca Ricci, Pina Lardo, Paola Addario Chieco, Antonio Stigliano

**Affiliations:** ^1^ Endocrinology, Department of Clinical and Molecular Medicine, Sant’Andrea University Hospital, Sapienza University of Rome, Rome, Italy; ^2^ Surgery, Department of Medical Surgical Sciences and Translational Medicine, Sant’Andrea University Hospital, Sapienza University of Rome, Rome, Italy; ^3^ General Surgery, Department of Surgical Sciences, Sant’Andrea University Hospital, Rome, Italy

**Keywords:** bilateral adrenal hemorrhage, adrenal insufficiency, abdominal sepsis, acute abdomen, orthopedic surgery, antiphospholipid syndrome, low molecular weight heparin

## Abstract

Adrenal hemorrhage is a rare, but important, diagnosis to recognize, in particular when there is involvement of both adrenal glands. Bilateral adrenal hemorrhage can in fact lead to adrenal insufficiency, with dramatic consequences if not promptly recognized and treated. It is normally caused by systemic conditions that lead to the vasoconstriction and thrombosis of the adrenal vein. Oftentimes, the clinical diagnosis of this condition can be very challenging, as its signs and symptoms are generalized and nonspecific (abdominal pain, nausea, and fatigue). Here, we present the cases of two patients admitted to the Emergency Department in 2016 and 2022 with acute abdominal pain, having recently undergone surgery and subsequently prescribed low-molecular-weight heparin. In both cases, laboratory results revealed neutrophilic leukocytosis and an unexplained anemia. Due to the persistence of abdominal pain despite medication, a CT scan was performed, showing an enlargement of both adrenal glands suggestive of bilateral adrenal hemorrhage. Adrenal function was tested that correlated with a diagnosis of adrenal insufficiency, and both patients were promptly treated with parenteral hydrocortisone as a result. On 5 years’ follow-up from the acute event, the second patient’s adrenal function had returned to normal, and he has not needed further adrenal replacement therapy; the first patient however demonstrated persistence of adrenal failure requiring replacement therapy. In this paper, through our experience and a literature analysis, we will aim to outline some clues to identify patients at potential risk of bilateral adrenal hemorrhage.

## 1 Introduction

Adrenal hemorrhage (AH) is a rare condition (1); most frequently, it is a consequence of an abdominal trauma, thus involving only one of the adrenal glands. Bilateral adrenal hemorrhage (BAH) represents 10% of all AHs and is usually caused by systemic conditions, such as surgery, septicemia, or coagulopathy. When BAH involves more than 90% of the adrenal cortex, potentially leading to acute adrenal insufficiency (AI), its consequences can be dramatic. AI is a life-threatening condition if not immediately recognized and treated (2). Conversely, unilateral AH is often asymptomatic.

The clinical diagnosis of BAH is challenging, with its signs and symptoms often vague and nonspecific, and thus can be difficult to frame clinically; its radiological features however are very typical and are usually diagnostic. Patients classically present with a variety of the following: abdominal pain, nausea, vomiting, anorexia, fatigue, tachycardia, hypotension, electrolyte imbalances (hyponatremia and hyperkalemia), hypoglycemia, and a drop in hemoglobin levels that cannot be otherwise explained (2). On occasion, the first clinical presentation is of acute AI with hemodynamic instability, with an associated mortality of 15% (3).

In this paper, we report the cases of two patients, both of whom presented to the Emergency Room with a nonspecific clinical picture similar to an acute abdomen and therefore went on to have an unenhanced CT scan that led to the diagnosis of BAH. For both patients, different risk factors (RFs) contributed to AH.

We will focus primarily on BAH, with it being a potential cause of acute AI. Many cases similar to ours are present in the current literature, however, unfortunately, these are isolated case reports lacking a systematic approach to the recognition and treatment of the different conditions leading to BAH. Through our experience and analysis of the literature, we have attempted to underline some clues to identify patients at potential risk for this condition and to create an intuitive approach to its diagnosis and follow-up, so that clinicians may promptly recognize and instigate the correct management.

## 2 Case description

### 2.1 Experience from case 1

A 52-year-old man presented to the Emergency Department (ED) with acute abdominal pain 11 days postoperatively. He had a recent history of acute colonic diverticulitis complicated by gut perforation and abdominal abscess, vertebral osteomyelitis treated with antibiotics, and radiological percutaneous drainage with pig tail, followed by left hemicolectomy. He was discharged from the hospital on the seventh day postoperatively with low-molecular-weight heparin (LMWH) 4,000 IU daily due to a medical history positive for smoking, diabetes, hypertension, myocardial infarction, left carotid occlusion, and colic diverticulosis complicated by several episodes of acute diverticulitis. In addition, he had a known left adrenal adenoma, which was never functionally characterized.

His previous regular medications included the following: acetylsalicylic acid 100 mg, clopidogrel 75 mg, bisoprolol 1.25 mg, pantoprazole 40 mg, and atorvastatin 40 mg.

In the ED, he had blood drawn and received an unenhanced abdominal CT scan, which showed acute inflammation and suspicion of a right colonic perforation, which was later unconfirmed at laparoscopy (negative operative findings).

**Figure 1:**
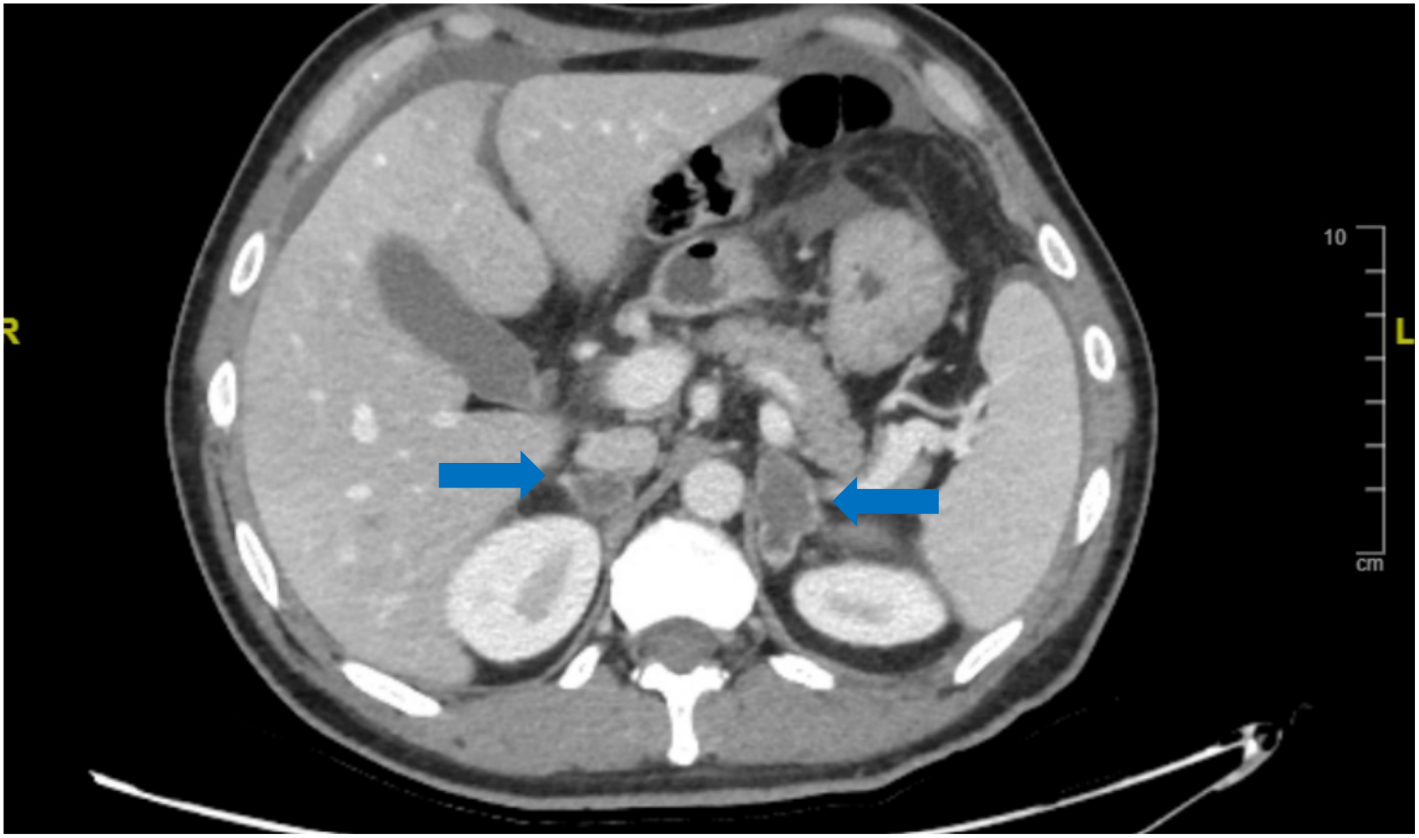
CT scan showing an acute increase of the dimensions (blue arrows) of both adrenal glands in case 1.

Due to the persistence of abdominal pain 24 h after the negative laparoscopy, a second contrast-enhanced CT scan was performed, which showed an acute increase of the dimensions of both adrenal glands, measuring 35 mm × 20 mm on the left and 21 mm × 23 mm on the right (**Figure 1**). In the previously performed cross-sectional images of the abdomen, bilateral adrenal hyperplasia was already visible, with a nodule of 18 mm in the medial portion of the left adrenal gland (increased in size over time). His blood tests showed a drop in hemoglobin levels (>1 g/dL in 24 h) with neutrophilic leukocytosis and an increase in C-reactive protein (CRP).

Blood pressure was 90/60 mmHg, and steroid profile and electrolytes were suggestive of AI (cortisol 77.3 nmol/l, aldosterone 16 pg/mL, Na 135 mmol/l, K 4.4 mmol/l). Increased Adrenocorticotropic hormone (ACTH) level (110 pg/mL) supported a diagnosis of adrenal failure (**Table 1**). Of note, plasma renin activity (PRA) 0.26 ng/mL/h was not as high as expected in AI, as the patient was taking a regular β-blocker prior. Adrenal replacement therapy was subsequently prescribed (hydrocortisone 15 mg in the morning upon awakening, 10 mg in the early afternoon, and fludrocortisone 0.1 mg per day). In addition, magnetic resonance imaging (MRI) confirmed the diagnosis of acute BAH.

**Table 1:** Baseline and dynamic hormonal examinations with imaging in both cases at presentation and at different times.

Case 1	Emergency Department	2 weeks after	1.5 months after		
Baseline cortisol (nmol/L)	77.3		161.1		
Cortisol at 60 min after ACTH test (nmol/L)			197.4		
Baseline ACTH (pg/mL)	110.0		66.4		
Adrenal imaging	CT scan: 3.5 cm × 2.0 cm on the left and 2.1 cm × 2.3 cm on the right	CT scan: 3.5 cm × 2.0 cm on the left and 2.1 cm × 2.3 cm on the right			
Case 2	Emergency Department	2 months after	12 months after	2 years after	5 year after
Baseline cortisol (nmol/L)	356.0	256.3	330.2	205.2	271.5
Cortisol at 60 min after ACTH test (nmol/L)		287.8	307.5		
Cortisol at 60 min after ACTH test (nmol/L)				387.4	505.8
Baseline ACTH (pg/mL)	115.6	120.9	111.7	46.5	44.7
Adrenal imaging	CT1 scan: imbibition of fat tissue around the left adrenal gland; CT2: 6.1 cm on the left and 5.1 cm on the right	CT: 4.1 cm on the left and 3.4 cm on the right		MRI: 7 mm on the right and 1.3 cm on the left	

Cortisol normal values: 165–508 nmol/L; ACTH normal values: 1.3–9.1 pg/mL; ACTH test 250 µg iv normal values: >500 nmol/L at 60 min.

Once the patient’s clinical condition had improved and blood tests normalized, he was discharged with a prescription for antibiotics, LMWH, and mineralocorticoid and glucocorticoid replacement therapy.

After 11 days, the patient returned to the ED with acute abdominal pain, nausea, vomiting, and diarrhea. Blood tests and abdominal examination were normal (Hb 14.9 g/dL, Na 144 mmol/l, K 4.2 mmol/l, glucose 142 mg/dL; normal renal and liver function, slightly positive CRP 0.85 mg/dL). Abdominal CT scan showed a thrombosis of the distal branches of the portal vein, causing ileal hypoperfusion, and confirmed the enlargement of adrenal glands. The need for emergency surgery was excluded, and the patient was discharged.

He presented to the adrenal outpatient clinic 1 month following diagnosis of AI, and his general condition on this occasion was good without any signs of adrenal crisis. Objective examination was unremarkable, blood pressure was 115/90 mmHg, and heart rate was 76 bpm. ACTH test 250 µg performed 45 days post-diagnosis showed persistence of AI (**Table 1**).

### 2.2 Experience from case 2

A 58-year-old man was referred to the ED after developing abdominal pain, fever, and severe fatigue. Seven days prior to undergoing bilateral hip replacement, he had been prescribed enoxaparin.His past medical history was unremarkable.

His laboratory tests showed leukocytosis with neutrophilia, a positive D-dimer and CRP, and normocytic anemia; potassium levels were normal while sodium was slightly low (135 mmol/L). Microbiological tests (blood, urine, and stool cultures) were negative as well as a hip X-ray performed in order to exclude abscess or hemorrhage in the surgical site.

A CT scan of the abdomen showed only some imbibition of the fat tissue surrounding the left adrenal gland.

**Figure 2:**
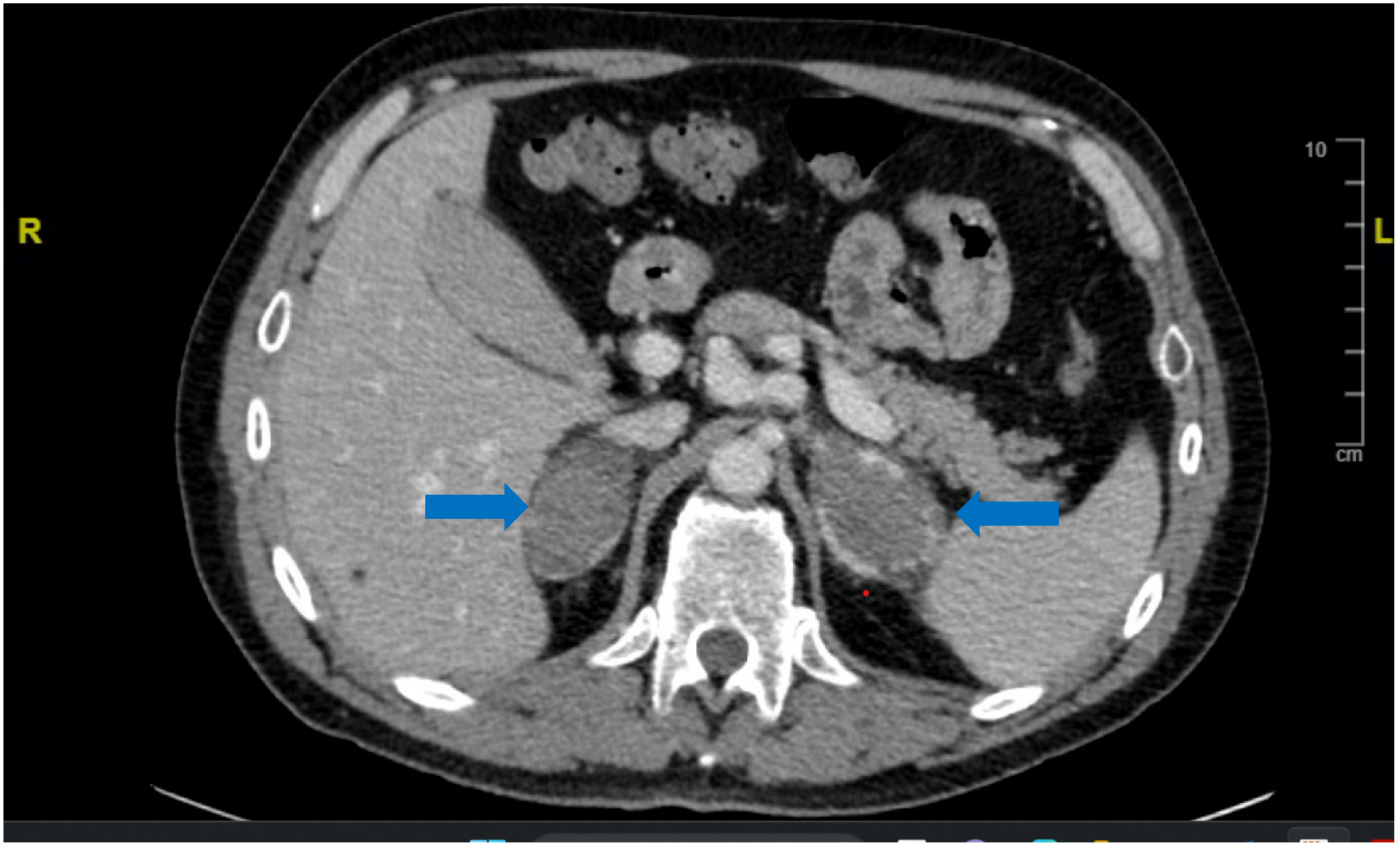
CT scan showing an increase of the size of both adrenals (blue arrows) with high density, suggestive for BAH, in case 2.

Despite antibiotic treatment, the patient’s condition did not improve, as he continued to develop worsening abdominal pain and asthenia. He became hypotensive (95/50 mmHg), and lab results confirmed a progressive drop in hemoglobin (1.6 g/dL in 7 days) with persisting mild hyponatremia. Whole-body CT scan was then repeated, and this showed a right pulmonary embolism associated with pulmonary infarction and a relevant increase in size of both adrenals (61 mm on the left and 51 mm on the right) with high radiodensity (50 Hounsfield units), which was suggestive for BAH (**Figure 2**).

Once blood had been drawn in order to assess adrenal hormones, hydrocortisone 100 mg intramuscular was administered, and then 75 mg of cortisone acetate (CA) per day orally thereafter. Following this, the patient’s symptoms improved rapidly, and there was a subsequent normalization of both arterial blood pressure (ABP) and sodium levels. Basal cortisol, aldosterone, and PRA were found to be normal, but ACTH remained high (115.6 pg/mL) (**Table 1**). In addition, he was prescribed rivaroxaban, and after approximately 1 month, the pulmonary embolism had completely reverted.

Coagulopathies and tuberculosis were ruled out as possible causes of AH [lupus anticoagulant (LAC), anticardiolipin antibodies, C and S protein, and Quantiferon test were all normal].

Patient was then discharged in good physical health, with a prescription for CA 75 mg per day.

Fifteen days post-discharge, he reported feeling well; blood pressure, electrolytes, and hemoglobin had normalized, and ACTH levels had reduced. He was then prescribed CA 37.5 mg per day.

These findings were in line with a CT scan demonstrating a reduction in sizes of both adrenal glands (41 mm on the left and 34 mm on the right).

Patient was reevaluated over time, and given his general condition and normal basal blood tests, progressive reduction of CA dosage was possible. In order to establish the need for corticosteroid replacement therapy, he underwent a 250-µg ACTH test after 2 months and then 1 year from the acute episode, and again after 2 years, which all revealed inadequate adrenal response to stimulus (**Table 1**). During follow-up, adrenal imaging was repeated, consistent with progressive normalization of adrenal dimensions. As a result of the SARS-CoV-2 pandemic, the patient was not reevaluated for a period of 2 years, during which time he autonomously decided to stop CA. He did not however experience adrenal crises.

Finally, 5 years on from the AH, when blood tests returned as normal, he performed another 250-µg ACTH test, which showed a complete reversion of AI, with no requirement for adrenal replacement therapy at all (**Table 1**).

## 3 Discussion

BAH is frequently related to one or more systemic RFs, such as surgery, septicemia, bleeding diathesis, or coagulopathy, i.e., systemic lupus erythematosus, antiphospholipid syndrome (APLS), anticoagulant therapy, or pregnancy (2).

Adrenal glands themselves are prone to bleeding as a result of the peculiar anatomy of their vascular system: the three adrenal arteries suddenly, in the zona reticularis, give origin to capillaries, which continue with a single vein. Therefore, if vasoconstriction and/or thrombotic occlusion of the adrenal vein occurs, the increased pressure in the capillaries can result in bleeding (2). The thrombotic occlusion of the adrenal vein is the main physio-pathological mechanism involved, being a consequence of one or more than one of the previously outlined RFs.

A sudden drop in arterial blood pressure and hemoglobin, together with hyponatremia (sodium <135 mmol/L), in the presence of one or more RFs, must raise the clinical suspicion of AH. As was the case in both of our patients, the diagnosis is usually only reached after an unenhanced CT scan of the abdomen is performed, which will show an acute increase in dimensions of both adrenal glands.

In these two cases, different RFs of AH can be identified; in particular, both had undergone major surgery and had then been prescribed LMWH.

Surgery represents a major stress on the body, associated with massive secretion of catecholamines and ACTH and, as a consequence, of cortisol. The high levels of these hormones in the adrenal vein leads to venoconstriction and platelet aggregation, with a resulting thrombosis of the adrenal vein.

Of note, the majority of surgeries reported in the literature as a cause for AH are orthopedic (**Table 2**); our second patient have had bilateral hip replacement surgery 7 days prior to the diagnosis of BAH. The higher frequency of BAH after orthopedic surgery may potentially be explained, at least in part, by the wide prescription of LMWH.

**Table 2:** Types of surgeries reported in patients with AH.

Types of surgery	Authors
Orthopedic	Kurtz 2007 Naka 2007 Best 2013 Ketha 2013 Mandanas 2013 Park 2015 Mudenha 2015 Wang 2019 Latina 2021 Patousis 2022
Abdominal	Rosenberger 2011 Kolinioti 2018 Wang 2019 Esparza Monzavi 2021
Cardiological	Vella 2001

In the literature, heparin exposure is the main reported RF for BAH (**Table 3**); the mechanisms through which heparin can lead to AH are not only related to its anticoagulant action. In a high percentage of cases, adrenal vein thrombosis is a consequence of heparin-induced thrombocytopenia (HIT) syndrome. Diagnosis of HIT can be reached through the identification of specific antibodies associated with this syndrome (heparin-PF4-IgG). Occasionally, however, no antibodies are observed (spontaneous HIT). A high prevalence of spontaneous HIT has been reported after knee and, less frequently, hip replacement surgery (4). HIT must be considered in the presence of nonspecific clinical symptoms, such as abdominal pain, nausea, and vomiting 4–12 days from anticoagulant therapy initiation (5). Almost 5% of patients treated with unfractionated heparin (UH) and nearly 1% of those treated with LMWH will develop HIT. This last figure is much higher following orthopedic surgery, reaching 4.8% (6), indicating orthopedic procedures themselves may increase the risk for HIT. Pulmonary embolism has been found to occur in 20% of patients with a diagnosis of HIT, as systemic coagulopathy is strongly associated with the condition (7); in all cases of AH in the context of HIT, thrombotic events in different organs can be found (4). BAH causing AI will happen in 4.8% of orthopedic patients exposed to UH, while only 0.6% of patients treated with LMWH will develop a hemorrhagic event associated with HIT (8).

**Table 3:** Types of anticoagulants reported in patients with AH.

Types of anticoagulants	Authors
Low-molecular-weight heparin	Kurtz 2007 Mandanas 2013 Park 2015 Kolinioti 2018 Wang 2019 Latina 2021 Patousis 2022
Unfractionated heparin	Rosenberger 2011 Ketha 2013 Jaafar 2015
Rivaroxaban	Ali 2018 Sheklabadi 2022
Apixaban	Sheklabadi 2022
Dabigatran	Best 2013
Coumarin derivatives	Naka 2007 Mudenha 2015 Khwaja 2017 Ali 2018 Kolinioti 2018 Bashari 2020

The importance of correctly identifying HIT as the cause of a hemorrhagic event, such as AH, precedes the need for an anticoagulant therapy, different to heparin, to treat this condition (4).

In both cases we reported, HIT-associated antibodies were not dosed, so we cannot make a certain diagnosis of HIT, neither definitely exclude it. In particular, patient 2 developed symptoms of AH (abdominal pain, fever, and fatigue) after 7 days from hip replacement surgery and the start of LMWH, with a concomitant diagnosis of pulmonary embolism. It must be noticed however that both patients had normal-to-high platelets, while thrombocytopenia is a typical finding in case of HIT.

Patient 1 not only was exposed to heparin but he was already taking dual antiaggregating therapy at home, increasing his global hemorrhagic risk.

The development of AH in the course of anticoagulant therapy though is a rare event; in the 20-year experience of Vella et al. (9) at the Mayo Clinic, only three patients of 141 total had AH while using anticoagulants, in particular during the first month of treatment.

Searching through the literature, we could only find five cases of novel oral anticoagulant-associated AH, all bilateral (10).

Sepsis is one of the oldest known RF of BAH; in 1911, the Waterhouse–Friderichsen syndrome was first described as a massive AH on a thrombotic basis associated with meningococcal sepsis. Nowadays, it is well established that a multitude of viruses and bacteria, different from *Neisseria meningitidis*, can cause this extremely dramatic syndrome. Even when AH and AI deriving from septicemia are immediately recognized and treated, mortality is very high, reaching nearly 15%. Moreover, AI in the context of sepsis is often irreversible (2).

Patient 1 had a recent history of acute diverticulitis with subsequent gut perforation and abdominal sepsis, which definitely contributed to the development of AH.

One of the most common RFs of BAH reported in literature is APLS (11). It is often diagnosed in patients who underwent major surgery and then started anticoagulant therapy with LMWH or in association with sepsis. The usual clinical picture of BAH due to this syndrome is again characterized by abdominal pain, anorexia, extreme fatigue, and a drop of hemoglobin levels otherwise unexplained; in both of our patients, these same symptoms were present; thus, APLS represented a plausible hypothesis, in particular for patient 2, who also developed pulmonary embolism. Indeed, specific antibodies of this syndrome have not been dosed. As for HIT, the utility of making the diagnosis of APLS is the need for an immediate anticoagulant therapy, to be continued lifelong (11). On the opposite, AI in this context is often reversible, so adrenal replacement therapy will only be temporary.

Another RF contributing to AH for patient 1 was the underlying left adrenal adenoma, which was already visible in CT scans he performed in the past and had increased in size over time.

It is not rare to detect previously unknown adrenal masses in patients with a diagnosis of AH. Sometimes, in the acute setting, when the bleeding is massive, those masses cannot be visible. This is one of the reasons why, during follow-up, it is essential to repeat abdominal imaging, specifically, enhanced CT scan or MRI, in order to exclude preexistent adrenal masses and to date and evaluate the remission of the hemorrhage itself (12). In the acute setting, unenhanced CT scan is the gold standard exam for the diagnosis of AH, given its extensive use in the ED, while enhanced CT and MRI usually represent second-level exams, performed mainly during the follow-up.

Adrenal lesions that more often bleed are metastases from other solid tumors; among adrenal primitive neoplasms, pheochromocytoma is the most frequent. More rarely, adrenocortical carcinoma (ACC) can be at the origin of the hemorrhage (2); this is a very aggressive tumor, so it must be immediately ruled out. A systematic review of the literature reported that 80% of bleeding adrenal masses are benign, in particular, adrenocortical adenomas and pheochromocytomas (13). Sometimes, even big myelolipomas can be a source of bleeding (2).

ACC and pheochromocytoma being the two adrenal lesions to be excluded first, ACTH, cortisol, Dehydroepiandrosterone sulfate (DHEAS), and urinary or plasmatic metanephrines should be evaluated during follow-up (13).

When BAH is suspected, because of the risk of AI, it is mandatory to test adrenal function. In the acute setting, the diagnosis of AI is made through an evaluation of plasmatic electrolytes together with basal adrenal hormones (cortisol, ACTH, aldosterone, and renin). The ACTH test will be performed only when the levels of basal hormones are doubtful, or during follow-up, if AI has been previously diagnosed, to monitor adrenal recovery (14).

Management of AH mainly depends on overall clinical conditions of the patient; sometimes, intensive medical treatment is required in order to face hemodynamic instability due to hemorrhagic shock, septicemia, and AI (13).

If there is a strong clinical suspicion of AI, immediately after the blood is drawn for adrenal hormones, even before having the results, parenteral hydrocortisone must be administered, followed by an oral therapy when AI is confirmed and patient condition is stable. A recent study establishes that continuous intravenous (CIV) hydrocortisone infusion is the safest administration route to manage the adrenal crisis (15). The authors demonstrated through a pharmacokinetic study that CIV infusion of 200 mg hydrocortisone over 24 h preceded by an initial bolus of 100 mg of the drug is able to maintain stable cortisol levels (15). Our patients have been treated differently: the first patient was not experiencing an adrenal crisis and he was clinically stable at the time of diagnosis, therefore, we opted for an oral therapy; the second one, on the opposite, received initial parenteral hydrocortisone in order to face hemodynamic instability.

Oral therapy of AI must follow the guidelines for primitive AI, being based on both glycoactive and mineraloactive adrenal hormones. Sometimes, aldosterone secretion can be preserved, even in case of BAH, with no need for mineraloactive replacement therapy, when aldosterone and renin levels are normal (16).

In the context of acute sepsis, the dosage of glycoactive hormones has to be increased according to the current protocols.

For both unilateral and bilateral AH, a conservative approach, based on a strict follow-up over time, must be the choice (2, 13) because adrenals can recover their normal function also after some years.

Follow-up is based on monitoring clinical conditions of the patient (i.e., ABP), blood tests (i.e., plasma electrolytes, glycemia, and creatinine), basal hormonal tests, and the repetition of the ACTH test over time, together with abdominal scans, in order to reassess adrenal function and monitoring the hematoma reabsorption. Patients should be addressed for follow-up to centers with high expertise in adrenal pathology.

Surgical treatment will be the choice in the presence of suspicious adrenal masses, such as pheochromocytoma and ACC, which need to be removed.

Back to our patients, in the first case, the ACTH test performed 45 days after the acute event showed a persistence of AI. Therefore, we expect as well the radiological persistence of the hematoma. Cross-sectional abdominal imaging scans will be performed every 6 months in order to measure the left adrenal lesion and the global adrenal size. Next, the ACTH test will be scheduled on the basis of basal hormonal levels and clinical features.

Patient 2 had different MRI scans of the abdomen during follow-up, which demonstrated a progressive reduction of adrenal sizes, compatible with hematoma resorption. In addition, the last ACTH test performed confirmed the reversibility of the AI, with a complete recovery of normal adrenal function after 5 years from the acute event, thus excluding a further need for adrenal replacement therapy.

The present paper takes its cue from clinical practice with the aim of focusing the attention of clinicians on the risk factors of AH. The following handbook contains some key points useful for the clinician to recognize AH and for the endocrinologist to treat it.

### 3.1 Learning points:

• Patients who experience hypotension or abdominal pain a short time after the start of any anticoagulant therapy must be screened for AH (9);

• AH must be suspected in patients who had surgery (in particular orthopedic surgery) and who do not improve as expected or who develop nausea, abdominal pain, and fever between the first and the second week after surgery (10);

• If a patient diagnosed with AH after a surgical operation has any thrombotic event in different organs, APLS has to be ruled out. Moreover, in all cases of AH with unknown etiology, screening with LAC and anticardiolipin antibodies is mandatory (17);

• Clinicians must be aware that BAH represents a dramatic consequence of abdominal sepsis, with a very high mortality even when it is promptly diagnosed and faced;

• Every patient with an acute bilateral enlargement of the adrenal glands must be evaluated for AI through the dosage of basal serum cortisol, ACTH, aldosterone, and renin, and, eventually, the ACTH test (18);

• Every patient with a suspicion of AI must be immediately treated even before hormonal results, and in case of an acute adrenal crisis, parenteral administration of hydrocortisone should be preferred;

• AH, both unilateral and bilateral, and the eventual AI associated are often reversible; thus, the treatment of choice should be a conservative approach based on a strict follow-up over time.

## Author contributions

MEA, SM, IB, RM, GR, FR, and PL collected surgery, endocrinologic, and radiological data. MEA wrote the draft article. AS and PAC performed data curation. AS acquired funding. All authors contributed to the article and approved the submitted version.

## References

[1] Mudenha ET Rathi M. Adrenal insufficiency due to the development of bilateral adrenal haemorrhage following hip replacement surgery. JRSM Ope (2015) 6(11):2054270415609837. doi: 10.1177/2054270415609837 PMC4641559 26673817

[2] Badawy M Gaballah AH Ganeshan D Abdelalziz A Remer EM Alsabbagh M. Adrenal hemorrhage and hemorrhagic masses; diagnostic workup and imaging findings. Br J Radiol (2021) 94(1127):20210753. doi: 10.1259/bjr.20210753 34464549 PMC8553189

[3] Wang L Wang XF Qin YC Chen J Shang CH Sun GF. Bilateral adrenal hemorrhage after hip arthroplasty: an initially misdiagnosed case. BMC Urol (2019) 19(1):106. doi: 10.1186/s12894-019-0536-7 31684918 PMC6829824

[4] Ketha S Smithedajkul P Vella A Pruthi R Wysokinski W McBane R. Adrenal haemorrhage due to heparin-induced thrombocytopenia. Thromb Haemost (2013) 109(4):669–75. doi: 10.1160/TH12-11-0865 23389301

[5] Rosenberger LH Smith PW Sawyer RG. Heparin-induced thrombocytopenia: an increasingly common cause of bilateral adrenal hemorrage. J Am Ger Soc (2011) 59:1157–8. doi: 10.1111/j.1532-5415.2011.03427.x 21668930

[6] Rosenberger LH Smith PW Sawyer RG Hanks JB Adams RB Hedrick TL. Bilateral adrenal hemorrhage: the unrecognized cause of hemodynamic collapse associated with heparin-induced thrombocytopenia. Crit Care Med (2011) 39(4):833–8. doi: 10.1097/CCM.0b013e318206d0eb PMC3101312 21242799

[7] Jaafar J Boehlen F Philippe J Nendaz M. Restoration of adrenal function after bilateral adrenal damage due to heparin-induced thrombocytopenia (HIT): a case report. J Med Case Rep (2015) 9:18. doi: 10.1186/1752-1947-9-18 25645253 PMC4417306

[8] Kurtz LE Yang S. Bilateral adrenal hemorrhage associated with heparin induced thrombocytopenia. Am J Hematol (2007) 82:493–4. doi: 10.1002/ajh.20884 17266058

[9] Vella A Nippoldt TB Morris JC 3rd. Adrenal hemorrhage: a 25-year experience at the Mayo Clinic. Mayo Clin Proc (2001) 76(2):161–8. doi: 10.1016/S0025-6196(11)63123-6 11213304

[10] McNicol RE Bradley A Griffin J Duncan G Eriksen CA Guthrie GJ. Post-operative bilateral adrenal haemorrhage: A case report. Int J Surg Case Rep (2014) 5(12):1145–7. doi: 10.1016/j.ijscr.2014.09.033 PMC4275802 25437659

[11] Kolinioti A Tsimaras M Stravodimos G Komporozos V. Acute adrenal insufficiency due to adrenal hemorrhage complicating colorectal surgery: Report of two cases and correlation with the antiphospholipid antibody syndrome. Int J Surg Case Rep (2018) 51:90–4. doi: 10.1016/j.ijscr.2018.07.034 PMC6111066 30145500

[12] Karwacka IM Obołończyk Ł Sworczak K. Adrenal hemorrhage: A single center experience and literature review. Adv Clin Exp Med (2018) 27(5):681–7. doi: 10.17219/acem/68897 29616752

[13] Ali A Singh G Balasubramanian SP. Acute non-traumatic adrenal haemorrhage-management, pathology and clinical outcomes. Gland Surg (2018) 7(5):428–32. doi: 10.21037/gs.2018.07.04 PMC6234242 30505763

[14] Bornstein SR Allolio B Arlt W Barthel A Don-Wauchope A Hammer GD. Diagnosis and treatment of primary adrenal insufficiency: an endocrine society clinical practice guideline. J Clin Endocrinol Metab (2016) 101(2):364–89. doi: 10.1210/jc.2015-1710 PMC4880116 26760044

[15] Prete A Taylor AE Bancos I Smith DJ Foster MA Kohler S. Prevention of adrenal crisis: cortisol responses to major stress compared to stress dose hydrocortisone delivery. J Clin Endocrinol Metab (2020) 105(7):2262–74. doi: 10.1210/clinem/dgaa133 PMC7241266 32170323

[16] Latina A Pellegrino M Chiefari A Lardo P Pia A Reimondo G. Reversibility of acute adrenal insufficiency after hip replacement: A case series. Endocr Metab Immune Disord Drug Targets (2021) 21(9):1669–72. doi: 10.2174/1871530320666201013155513 33050871

[17] Bansal R Nath PV Hoang TD Shakir MKM. adrenal insufficiency secondary to bilateral adrenal hemorrhage associated with antiphospholipid syndrome. AACE Clin Case Rep (2019) 6(2):e65–9. doi: 10.4158/ACCR-2019-0376 PMC7282157 32524013

[18] Naka N Takenaka S Nanno K Moriguchi Y Chun BM Sonoda S. Acute adrenal crisis after orthopedic surgery for pathologic fracture. World J Surg Oncol (2007) 5:27. doi: 10.1186/1477-7819-5-27 17338824 PMC1821329

